# Quantification of dendritic cell subsets in human thymus tissues of various ages

**DOI:** 10.1186/s12979-021-00255-8

**Published:** 2021-11-18

**Authors:** Yan Li, Pei Chen, Hao Huang, Huiyu Feng, Hao Ran, Weibin Liu

**Affiliations:** 1 Department of Neurosurgical Intensive Care Unit, The First Affiliated Hospital of Sun Yat-sen University, Guangzhou, 510080 China; 2 Department of Neurology, National Key Clinical Department and Key Discipline of Neurology, The First Affiliated Hospital, Sun Yat-sen University, Guangzhou, 510080 China; 3 Department of Neurology, The First People’s Hospital of Nanning, Nanning, 530022 China; 4 School of Pharmaceutical Sciences, Sun Yat-Sen University, Guangzhou, 510006 China

**Keywords:** Dendritic cell, Thymus, Aging, Plasmacytoid dendritic cell, Myeloid dendritic cell

## Abstract

**Background:**

Dendritic cells (DCs) in the thymus are involved in central tolerance formation, but they also have other functions in the thymus, such as pathogen recognition. The density changes of human thymic DCs have been hardly investigated. In this study, human thymus samples of various ages were collected for tissue sectioning and staining. The thymic cortex and medulla area as well as the densities of various subsets of thymic DCs were calculated.

**Results:**

All common DC subsets were found in the human thymus of various ages. Most DCs had accumulated in the human thymic epithelial space, especially the medulla. We also found that the human thymic cortex had atrophied relatively faster than the medulla, which led to a gradual increase of the area ratio of the medulla to cortex with the increase of age. The densities of DC subsets in the human thymus showed various changes with increasing age, which contributed to the composition changes of DC subsets. The density of plasmacytoid DCs (pDCs) in the human thymus had increased gradually with aging, which suggested that pDCs plays another essential role in the thymus in addition to central tolerance.

**Conclusions:**

Inconsistent with the shrinking of the epithelial space in the thymus, the densities of DC subsets in the epithelial space of the thymus are maintained at a constant level with aging to preserve highly efficient autoreactive thymocyte screening. An increasing density of the thymic pDCs with aging implies an extra function of DCs in the thymus beyond central tolerance.

**Supplementary Information:**

The online version contains supplementary material available at 10.1186/s12979-021-00255-8.

## Background

The thymus is the central lymphoid organ in which T cells develop and mature. During this process, the body executes a series of elaborate mechanisms to remove thymocytes that cannot recognize their own human leukocyte antigen (HLA) or autoreactive thymocytes and finally outputs naive T cells and natural regulatory T cells. This is the formation of central tolerance, which is a critical component of immune tolerance [1, 2]. Disruption of central tolerance often leads to autoimmune diseases, such as myasthenia gravis, whose pathogeneses are closely related to the thymus [3–5].

Dendritic cells (DCs) and thymic epithelial cells are the main components for thymocyte screening in the thymus [2, 6, 7]. Currently, it is believed that there are two origins of DCs in the thymus. Some DCs are derived from precursor cells of DCs or dendritic cells in the blood, while others differentiate from progenitor cells that migrate from the bloodstream to the thymus [8–10]. Regardless of the origin, DCs in the thymus participate in central tolerance formation and are mainly concentrated in the medulla. DCs in the medulla collect various common self-antigens and tissue-restricted antigens expressed by thymic epithelial cells. DCs migrate from blood and carry peripheral antigens to the thymus to enrich the central tolerance self-antigen library [7].

Similar to DCs in other tissues, DCs in the thymus are divided into plasmacytoid dendritic cells (pDCs) and myeloid dendritic cells (mDCs; also called conventional dendritic cells) [11]. mDCs can be further divided into CD1c-positive and CD141-positive mDCs [12, 13].

Although the specific function of DCs determines their role in the immune response, the subset composition of thymic DCs also modulates the generation of thymic Tregs [14]. Therefore, it is vital to understand the role of DCs in thymus tissue in detail and define the distribution and quantitative characteristics of the two main DC subsets in the human thymus. Additionally, the human thymus atrophies gradually with age, especially after puberty. It is unclear which changes occur in the distribution and density of DCs in the thymic epithelial space while the thymus atrophies. Studying the composition of DC subsets in the human thymus and their density changes with aging might help us to understand the role of DCs in the human thymus.

## Results

### Dendritic cell subsets and morphological characteristics in the human thymus

**Fig. 1:**
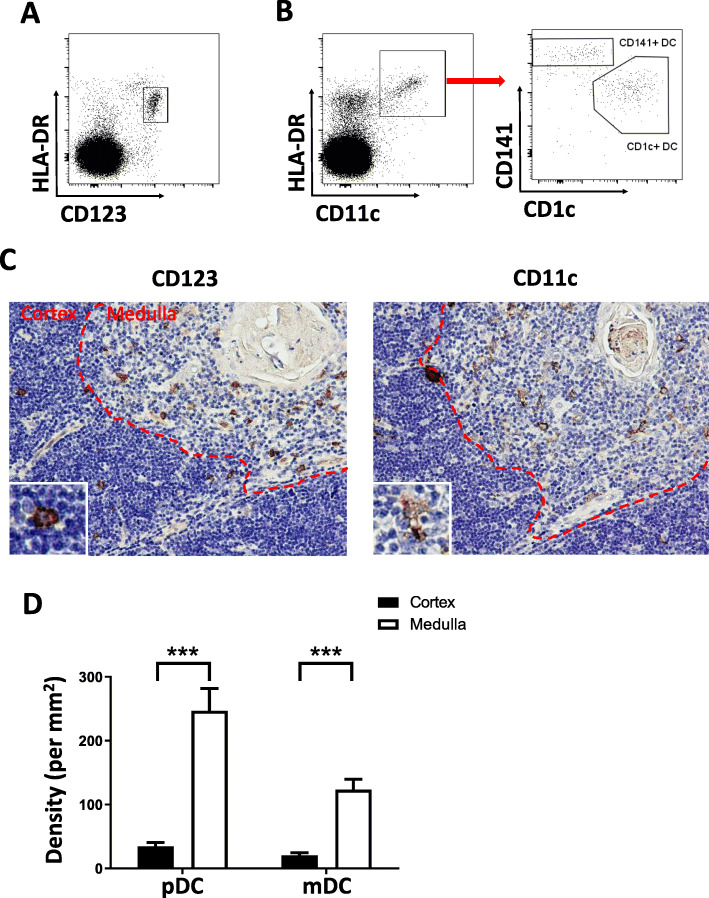
Dendritic cell subsets and morphological characteristics in the human thymus. **A**, other kinds of cells in the thymus were excluded by Lineage cocktail antibodies and pDCs (HLA-DR positive and CD123 positive, in the box) were labeled with HLA-DR and CD123. **B**, to exclude other types of cells in the thymus, mDCs were labeled with HLA-DR and CD11c (HLA-DR positive CD11c positive, in the box). MDCs could be further divided into CD141 positive DCs and CD1c positive DC by CD1c and CD141. **C**, in immunohistochemical staining, CD123 was used to mark pDCs (brown, granular). CD11c was used to mark mDCs (brown, dendritic) in the thymus. The two subsets were mainly distributed in the thymus medulla. Images are in 200×, and small images in the bottom left are enlarged to show the appearance of different DC subsets. **D**, pDCs, and mDCs densities in the thymic medulla and cortex were calculated and compared. ***, *P* < 0.001

Thymus tissue was digested into a single cell suspension and dendritic cells in the thymus were analyzed by flow cytometry. We used a lineage cocktail of antibodies to exclude thymocytes, T cells, B cells, NK cells, and macrophages. Cells that were negative for the lineage cocktail and positive for HLA-DR were selected for further analysis. Plasmacytoid DCs (pDCs) were labeled by CD123 (Fig. 1A). Another dendritic cell subset, myeloid DCs (mDCs) (Fig. 1B), was labeled by CD11c and further divided into CD1c- and CD141-positive DCs (Fig. 1B). Using CD123 and CD11c as markers in immunohistochemistry, the two subsets of DCs were identified in thymus tissue (Fig. 1C). pDCs were elliptic and granular, while mDCs were typical dendrite-like with irregular shapes and many projections. Although mDCs were further labeled by CD1c and CD141 in multicolor flow cytometry, these two markers had poor specificity when used in immunohistochemistry. Both pDCs and mDCs were mainly distributed in the thymus medulla (Fig. 1D) and the density of pDCs in the thymus was higher than that of mDCs (Fig. 1D).

### The ratio between the medulla and cortex area in the human thymus increases with age

**Fig. 2:**
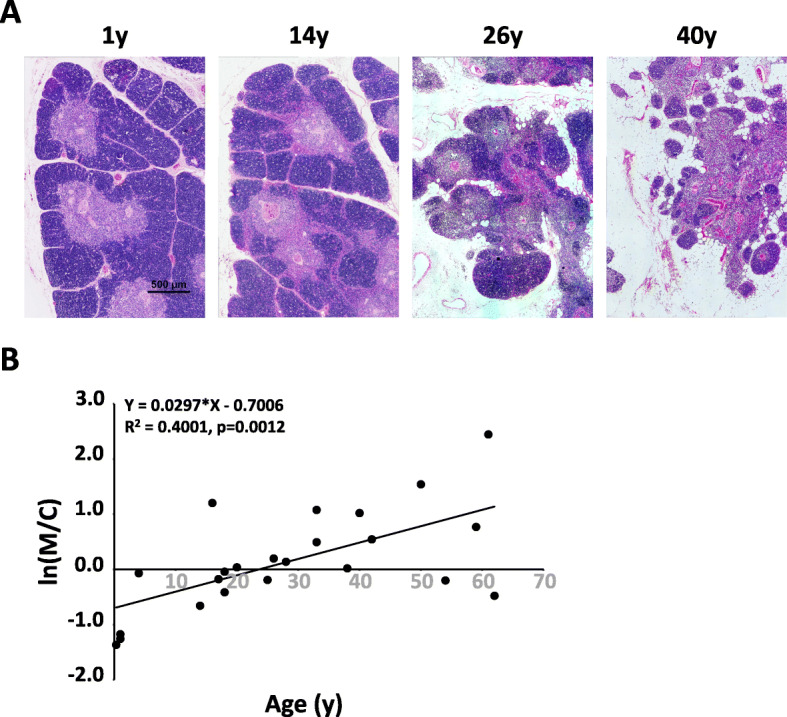
The ratio between the medulla and cortex area in the human thymus increases with age. **A**, HE staining of thymus tissue slides with different ages showed medulla, cortex, and gradually increasing adipose tissue in the thymus. **B**, the ratio of thymic medulla area to cortical area (M/C) and the relationship between M/C and ages, the variable x in the formula is ln(M/C), *n* = 23

Normal thymuses of various ages were collected for HE staining. Four specific age groups (1 year: infants; 14 years: adolescents; 26 years: young adults, 40 years: middle aged) were selected to represent the characteristics of thymus changes with age (Fig. 2A). The thymus atrophies gradually with increasing age and rapidly atrophies after puberty. The thymus is replaced mainly by adipose tissue by adulthood, but a small amount of the thymus medulla and cortex remains. Although the thymus gradually atrophies with age and is replaced by adipose tissue, the atrophy degree of the thymic cortex and medulla is not uniform (Fig. 2A). We calculated the area of the medulla and cortex in stained thymus tissues and calculated the ratio (AreaM/AreaC, M/C) between the medulla (AreaM) and cortical (AreaC) to establish the relationship between the ratio and age. With aging, the M/C ratio increased gradually and significantly (Fig. 2B), which suggested that the thymic cortex atrophied more rapidly than the medulla.

### mDC density does not change significantly with aging, while pDCs increase gradually with aging of the human thymus

**Fig. 3:**
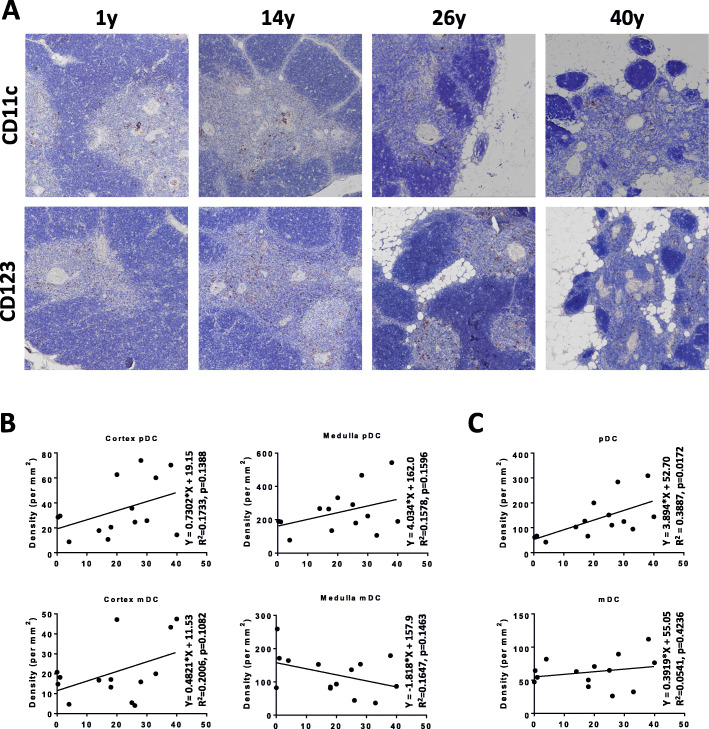
PDCs increase gradually with the aging of the human thymus. **A**, IHC was used to present the mDCs and pDCs in human thymic tissue from different ages (1, 14, 26, and 40-years). CD11c was used to label mDCs and CD123 to label pDCs. Images are in 100×. **B**, the densities of pDCs and mDCs in the medulla and cortex were calculated respectively to show their changes with increasing age. **C**, the density of pDCs and mDCs in the medulla and cortex showed a changing trend with ageing, and the pDCs present a significant increase with ageing. (pDC group, *n* = 14; mDC group, *n* = 14)

Using CD123 and CD11c to mark DCs in thymus tissues of various ages (Fig. 3A), we found changes in pDC and mDC densities. With the increase in age, most of the thymic medulla and cortex was replaced by fat components in the typical 40-year-old thymus, which was not suitable for staining and density calculation of DCs. Therefore, normal thymus tissue over 40 years of age was not included in the analysis. By analyzing the density of the two DC subsets in the thymic medulla and cortex, we found that the densities of DC subsets in the thymic medulla and cortex had changed with a certain tendency. The density of pDCs and mDCs in the medulla was significantly higher than that in the cortex regardless of age. As age increased, the density of pDCs in the thymic medulla and cortex had increased gradually. However, the density of mDCs in the medulla and cortex showed the opposite trend with the increase in age. We observed a gradual decrease in the medulla and a gradual increase in the cortex (Fig. 3B). Combining the density of the two DC subsets in the medulla and cortex, we found that the density of pDCs had increased gradually with age, thymus atrophy, and the increased proportion of the medulla (Fig. 3C). Simultaneously, the density of mDCs had also increased, but did not change significantly (Fig. 3C).

### Composition of DC subsets in the human thymus changes with age

**Fig. 4:**
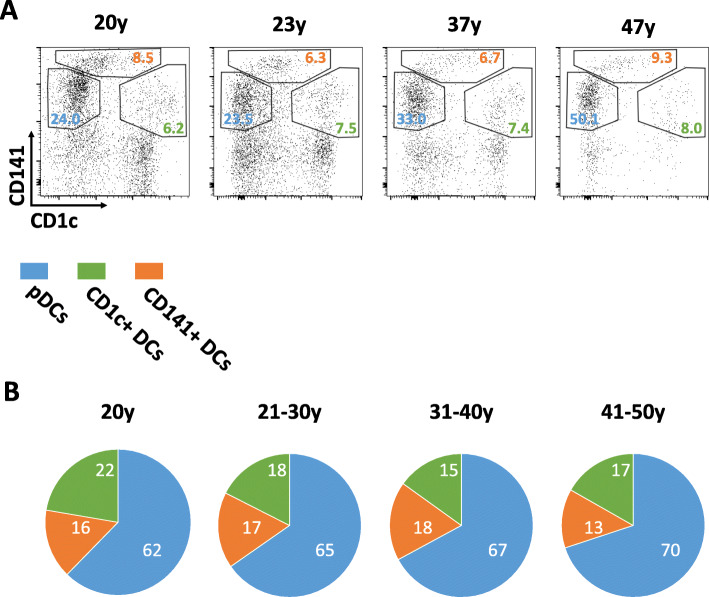
The composition of DC subsets in the human thymus with different ages. **A**, the typical compositions of each DC subsets in the human thymus with different ages were present in flow cytometry. The cells were gated on Lineage negative and HLA-DR positive cells from the single-cell suspension made from the digestion of thymus tissue. Three DC subsets could be separated into three different groups by markers CD1c and CD141. **B**, the percentages of each DC subsets were calculated by flow cytometry. Four age ranges were grouped. The means of percentages in different age ranges were present in the pie chart. (20y, *n* = 1; 21-30y, *n* = 3; 31-40y, *n* = 4; 41-50y, *n* = 2)

We used immunohistochemistry (IHC) to determine the densities of pDCs and mDCs. By calculating the percentage of each DC subset, we found that pDCs occupied an increasing proportion in the thymus with ageing. However, because of the marker limitation, it was difficult to label subsets of mDCs, which included CD1c + DCs and CD141+ DCs, by IHC. As shown in Fig. 1, distinguishing subsets of mDCs by flow cytometry was easy. Therefore, we used flow cytometry to determine the composition of human thymic DCs that included mDC subsets. IHC showed that pDCs comprised most thymic DCs. A growing percentage of pDCs was found with the increase in age from ~ 60% at 20 years of age to ~ 75% in 47-year-olds (Fig. 4A). To reveal the trend of the composition of thymic DC subsets with age, we divided the thymus samples into four age groups: 20, 21–30, 31–40, and 41–50 years. As age increased, the percentage of pDCs among human thymic DCs grew steadily from 62 to 70%. Correspondingly, the percentage of mDCs had decreased. However, CD1c + and CD141+ DCs had similar proportions among mDCs. Each mDC subset rate did not change obviously (Fig. 4B).

## Discussion

In 2010, Ziegler-Heitbrock, L et al. declared a consensus on a flow cytometry panel for human peripheral blood DCs labeling. In this consensus, Linage negative HLA-DR positive CD11c positive for mDCs, or Linage negative HLA-DR positive BDCA2 positive for pDCs [15]. There is no uniform labeling method for DCs in different organs, but Linage, HLA-DR, BDCA2 (or CD123), CD1c, CD141 and CD11c are widely used to mark DCs in human tissues [16]. However, immunohistochemistry cannot identify DCs by multiple staining as flow cytometry does. Currently, CD11c, CD123 and BDCA2 are commonly used to label DCs in immunohistochemistry. In several previous studies, CD86, DC-LAMP and other markers were also used to mark DCs in tissues [11, 17, 18]. Although CD11c and CD123 are not exclusive markers, these two markers can be used alone to specifically mark two different dendritic cell subsets, as our results showed.

Thymus comprises the following two compartments: the thymic epithelial space – the site of thymopoiesis occurs – and the perivascular space. The thymic epithelial space comprises the well-known thymic cortex and medulla. Thymus atrophy means the shrinking of the thymus with ageing in mice; however, in humans, thymus atrophy refers to the decrease in size of the thymic epithelial space with age. Therefore, we focused on the thymic cortex and medulla in this histological research.

Thymus atrophy is a normal physiological process, and the most widely accepted hypothesis for its existence is the “disposable-soma” theory. This theory hypothesizes that the body supplies and distributed energy to each organ depending on their importance in the continuation of future generations [19]. Based on this theory, Andrew George and Mary Ritter further explained the significance of thymus atrophy [20]. They surmised that constantly responding to foreign and self-antigens allows the immune system to gradually build a reliable memory T cell repertoire for immune defense. Continuous production of great amount of new naive T cells to further enriches the repertoire thus becomes unnecessary. The production of T cells requires energy and resources, and 95% thymocytes are eliminated during the positive and negative selection in thymus. Therefore, the thymus function is lost and the organ undergoes atrophy.

We stained human thymus tissue to further focus on the two compartments of the thymic epithelial space, that is, thymic cortex and medulla. These structures play different roles in the development of thymocytes [21]. In the cortex, thymocytes that express a T-cell receptor with sufficient affinity for HLA-peptide complexes survive and further differentiate through the process of positive selection. In the medulla, thymocytes are screened for reactivity against nonubiquitous self-antigens primarily [22]. Thus, the medulla needs to retain its function during the thymus atrophy with age. A relatively slow involution might prevent the release of self-reactive naive T cells. Consistent with this speculation, the analysis of the cortex and medulla of thymic tissue sections of different ages showed that the medulla had a lower involution rate as indicated by the increase in the area ratio of the medulla to the cortex.

Similar to the roles of thymic epithelial cells, DCs in human thymus also contribute to central tolerance by inducing the production of naive T cells and nTregs [23]. Both mDCs and pDCs induce central tolerance in thymus, and mDC is more efficient [24]. The subsets mDC1 (CD8α and XCR1 positive in mice, CD141 positive in human) and mDC2 (CD11b and Sirpα/CD172a positive in mice, CD1c and CD11b positive in human) in mice also display different efficacy in the induction of central tolerance [25]. pDCs may be better at transporting special antigens from peripheral tissues (other than the thymus) to induce negative selection [26].

Because of the potential for improving immunity in elderly individuals, restoration of the thymus function is an ascendant research field. However, there were few studies on age-dependent changes in the density of dendritic cells in the thymic epithelial space, and most of these studies were conducted in mice [25, 27, 28]. Considering the limitation of flow cytometry to distinguish cortex and medulla, in this study, we preferred histological methods to analyze the density changes in DCs in the cortex and medulla. Our results showed that the mDCs density in human thymus did not change significantly with aging, whereas the density of pDCs gradually increased. The results are consistent with those in mice. Changes in thymic DCs in mice with age suggested that the number of mDCs was different in each subset, with mDC1 gradually decreasing and mDC2 gradually increasing with age. pDCs gradually increased with age [25]. However, previous studies in humans showed that the percentage of thymic DCs decreased with aging [29, 30]. Nakahama et al. [29] also studied the number of DCs in the thymic medulla at different ages using histochemical markers and cell morphology but did not distinguish pDCs and mDCs, and the DC markers they used (S100 and LN-2) are currently not typically selected. Varas et al. [30] used the method of enrichment and sorting to study the proportion of all DCs in human thymus at different ages, but the age range of the study was only newborn to 10 years.

The significance of changes in the density of DCs with ageing remains currently unclear. The loss of thymopoietic space with age is accompanied by a corresponding decrease in the thymic output of naive T cells and nTregs; however, relatively conserved medulla and the constant density of mDCs in this region retain significant function for negative selection to prevent autoimmunity. In addition, the density of pDCs increased with ageing, which suggests that pDCs play other vital roles even as the thymus undergoes atrophy. This role may not involve the central tolerance induction, but the specific role needs to be further studied.

## Conclusions

The relative proportion of cortex to medulla structures in human thymus tissue changes with age, and the relative retention of medulla might indicate the importance of central tolerance. The density of plasmacytoid dendritic cells increases gradually, which suggests that some of the functions they perform in human thymus become relatively more important with ageing.

## Methods

### Sample collection and processing

Thymus samples were collected from patients that underwent corrective cardiology surgery at the Cardiac Surgery Department of the First Affiliated Hospital of Sun Yat-sen University and Guangdong Provincial People’s Hospital from June to November 2014. The normal thymus samples for histology were obtained from patients aged 4 months to 62 years (*n* = 23). Samples for flow cytometry were collected in 2016 and 2021 (*n* = 10). The surgically removed specimens were immediately placed in a specimen transfer box for laboratory processing. All thymus specimens were collected, transferred, and treated in accordance with the regulations of the Ethics Committee of the First Affiliated Hospital of Sun Yat-sen University and under its supervision.

### Thymus sampling

In thymus samples under 15 years of age, the involuted parts of the thymus were very small. Therefore, any part of the thymus from patients under adolescence were represented of the whole thymus in this age. For thymus samples from 15 years of age and above, uneven contents in the involuted thymus had a significant influence on the sampling reliability. Furthermore, only two or three 15 × 15 × 10 mm sections were collected from a resected thymus. To choose a thymus section for reliability when interpreting the events in the aging thymus, some rules were followed when sampling. First, the involution of the thymus followed a fixed mode as descripted by Haynes et al [31]. Second, replacement of the epithelial space by adipose in thymus began from the peripheral part of the thymus. Therefore, we chose to sample tissue from the inside parts of the thymus and not peripheral parts.

We also calculated cortex and medulla areas for all ages after hematoxylin and eosin (HE) staining. However, we only calculated the density of thymic DCs from less than 40 years of age, which avoided a sampling bias because of the excessively scattered epithelial space seen in the thymus from older than 40 years. When we calculated the numbers of DCs and the cortex and medulla areas, we scanned the whole section to avoid sampling bias.

### Flow cytometry

Thymus samples were cut into approximately 1 mm^3^ pieces and digested in RPMI medium, Collagenase D (1 mg/mL), Collagenase IV (1.5 mg/mL), and DNase I (0.05 mg/mL) in an incubator with 5% CO_2_ for 45 min. The tissue blocks were suspended by shaking every 15 min. After digestion, the tissue blocks were dissociated by pipetting. The sample was passed through a 70-μm filter to prepare a single cell suspension for flow cytometry. A lineage cocktail of antibodies (anti-CD3, −CD14, −CD16, −CD19, −CD20, and -CD56 antibodies, 348,701, Biolegend) and antibodies against HLA-DR (327,013, Biolegend), CD11c (301,641, Biolegend), and CD123 (306,015, Biolegend) were added to the single cell suspension, followed by incubation for 30 min at 4 °C for staining. For specific staining methods, please refer to a previous study [32]. After staining, the single cells of the thymus samples were analyzed by flow cytometry on a CytoFLEX Flow Cytometer (Beckman Coulter). Data were analyzed using Flowjo Vx software (TreeStar software).

### Tissue section staining and immunohistochemistry

The collected thymus samples were cut into 15 × 15 × 5 mm pieces and fixed in 4% paraformaldehyde (Sigma-Aldrich) for 24 h at room temperature. Selected tissue samples were trimmed for dehydration and embedding in paraffin. After embedding, 4-μm-thick sections of tissue were prepared and mounted on slides. After dewaxing and hydration, the tissue sections were stained with hematoxylin and eosin (BASO) by following a typical procedure. For immunohistochemistry, antigen retrieval was performed on rehydrated tissue by boiling the sections in antigen retrieval Citra Solution (Gene tech). Sections were quenched for 30 min at room temperature in a 3% H_2_O_2_ solution (Gene tech), followed by incubation with anti-CD11c (ab52632, Abcam) or -CD123 (GT2136, Gene tech) primary antibodies overnight at 4 °C. Staining with biotinylated secondary antibody was performed for 1 h at room temperature. Staining was developed using an ABC kit (Gene tech) and DAB kit (Gene tech), and counterstaining was conducted with hematoxylin (BASO).

### Calculation of thymic medulla and cortex areas and DC densities

A Nikon ECLIPSE Ni-U series microscope was used to obtain images of HE and IHC staining in thymus tissue sections. Images of whole tissue sections were analyzed by image synthesis software (NIS-Elements D). HE staining was used to calculate the area of the medulla and cortex of thymus tissue. The cortex and medulla of the thymus tissue were circled and the actual size was calculated by NIS-Elements D software. IHC staining was used to calculate the density of dendritic cells. Using NIS-Elements D, the specific numbers of dendritic cells in the cortex and medulla were counted and divided by the area of the cortex or medulla (mm^2^) to obtain the average density of dendritic cells in the cortex or medulla (per mm^2^).

### Statistical analysis

The unpaired parametric two-tailed Student’s t-test was used to calculate statistical significance between two groups. The correlation between age and the area ratio of the medulla to cortex (the ratio was converted to a logarithm) was fitted by linear regression. Correlation analysis of the age and density of dendritic cells in the thymus and cortex employed linear regression fitting.

## Supplementary Information

**Additional file 1: **Immunofluorescence was used to confirm CD11c and CD123 as the single-specific marker for mDCs and pDCs, respectively. A, CD74 (HLA-DR) and CD123 co-staining; B, CD123 and CD11c co-staining; C, CD74 and CD11c co-staining.

## Data Availability

The datasets used and/or analyzed during the current study are available from the corresponding author on reasonable request.

## Abbreviations

BDCA: blood dendritic cell antigen
DCs: dendritic cells
HE: hematoxylin and eosin
HLA: human leukocyte antigen
IHC: immunohistochemistry
mDCs: myeloid dendritic cells
MG: myasthenia gravis
pDCs: plasmacytoid dendritic cells
