# Supplementary material for: Quantification of dendritic cell subsets in human thymus tissues of various ages
Source: Immun Ageing. 2021 Nov 18;18:44. doi: 10.1186/s12979-021-00255-8 (PMC8600781; doi:10.1186/s12979-021-00255-8)

- Supplementary Figure 1 Immunofluorescence was used to confirm CD11c and CD123 as the single-specific marker for mDCs and pDCs, respectively. A, CD74 (HLA-DR) and CD123 co-staining; B, CD123 and CD11c co-staining; C, CD74 and CD11c co-staining.

Supplementary Figure 1

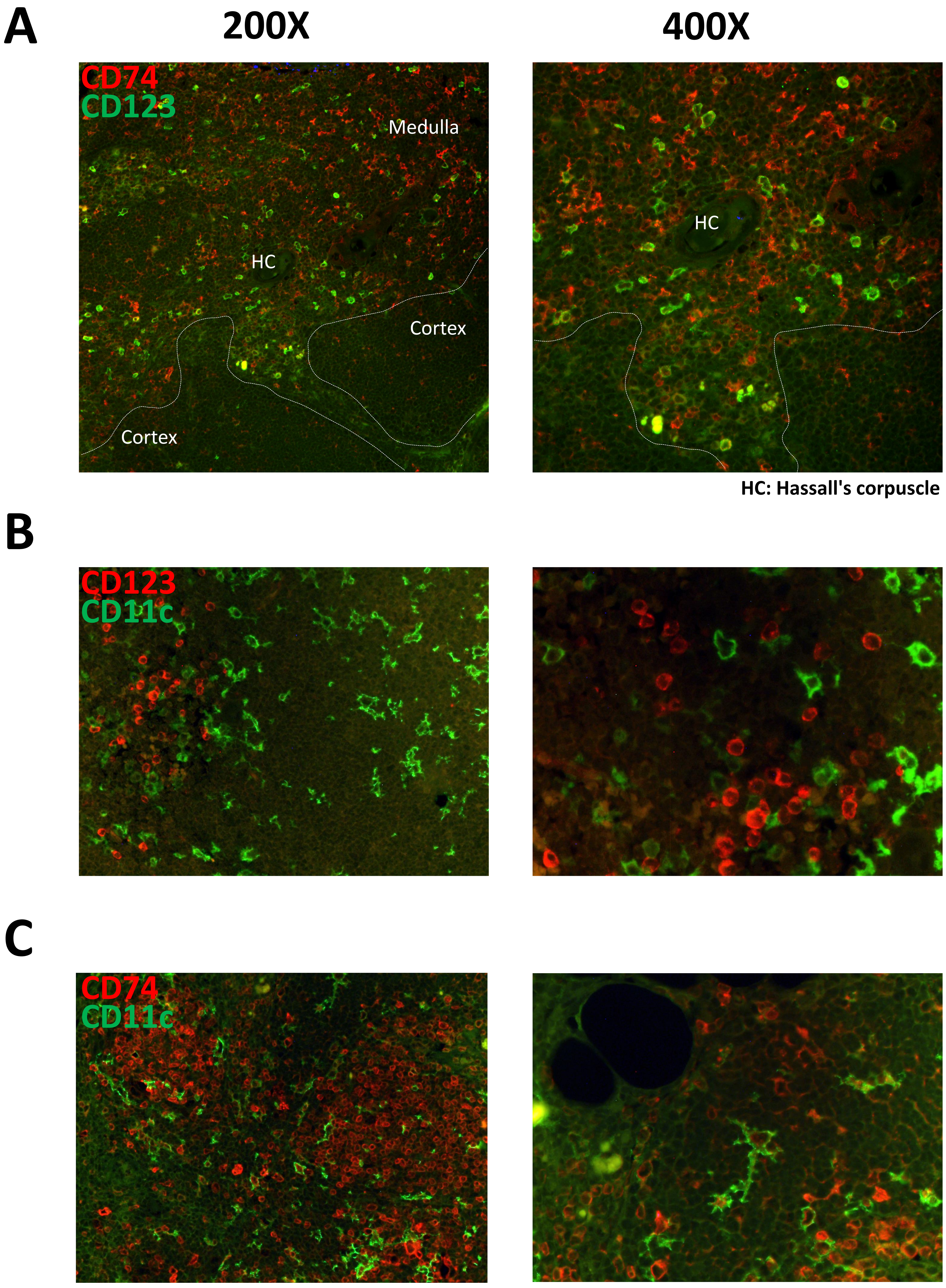

Supplement: Supplementary file 1 — Additional file 1: Immunofluorescence was used to confirm CD11c and CD123 as the single-specific marker for mDCs and pDCs, respectively. A, CD74 (HLA-DR) and CD123 co-staining; B, CD123 and CD11c co-staining; C, CD74 and CD11c co-staining. [file 12979_2021_255_MOESM1_ESM.pdf]
